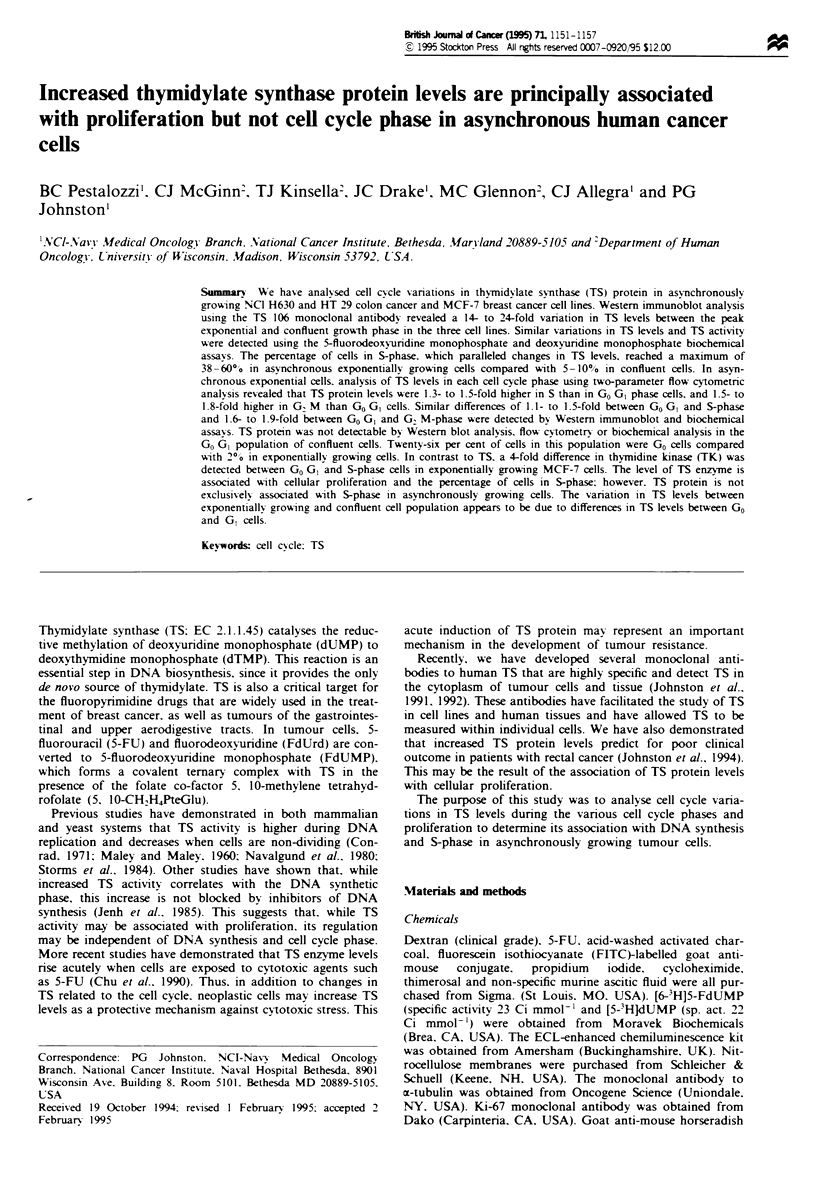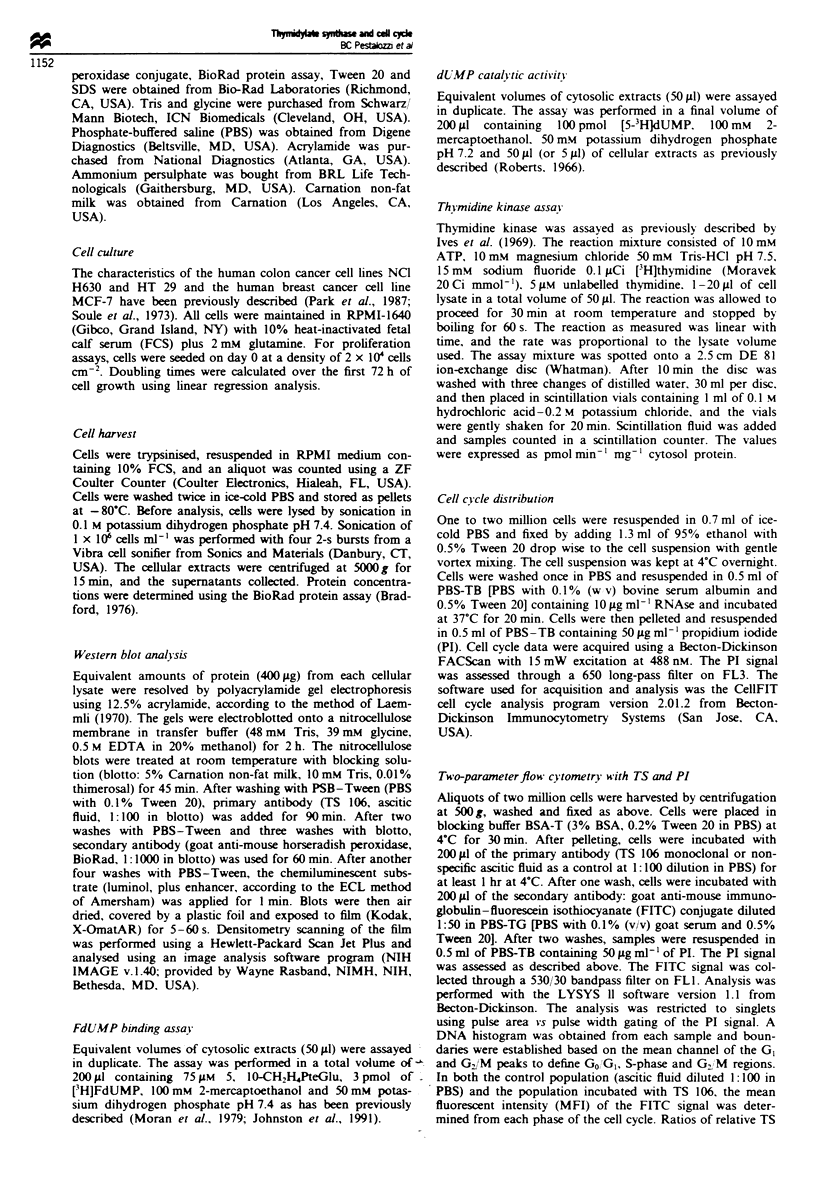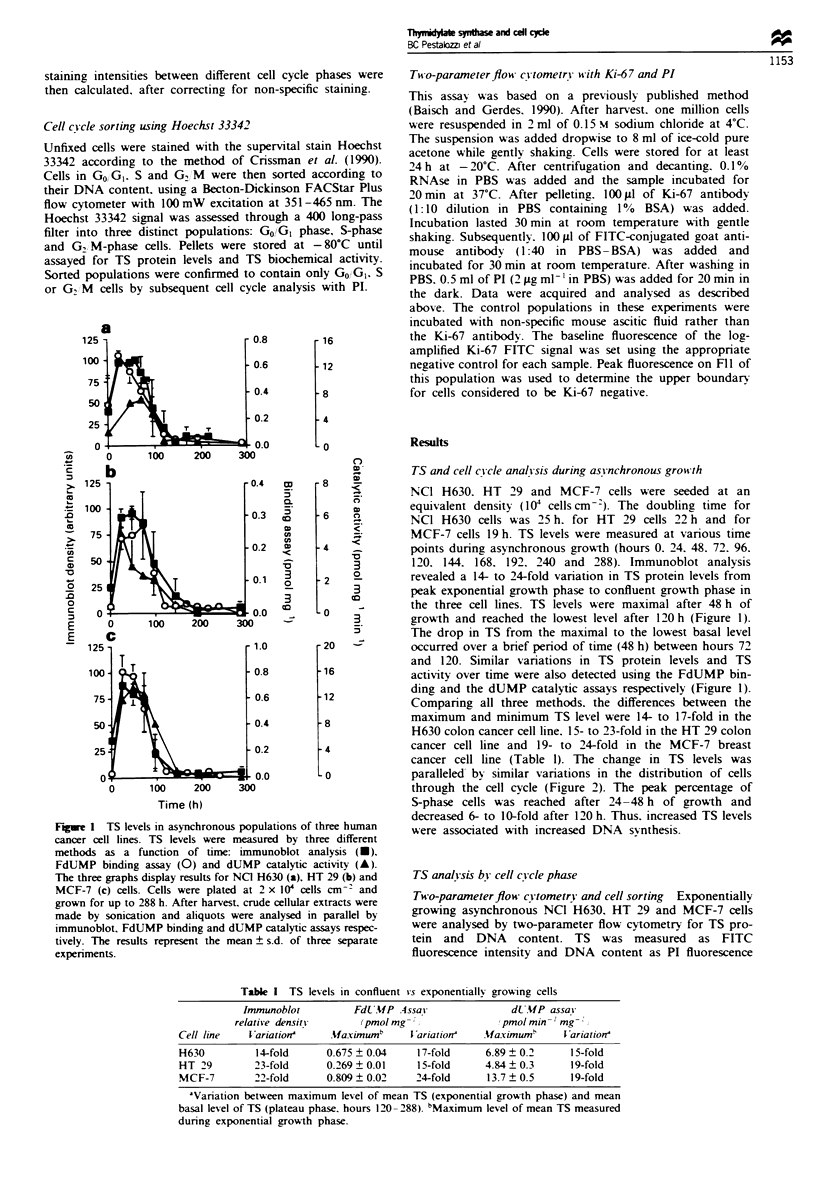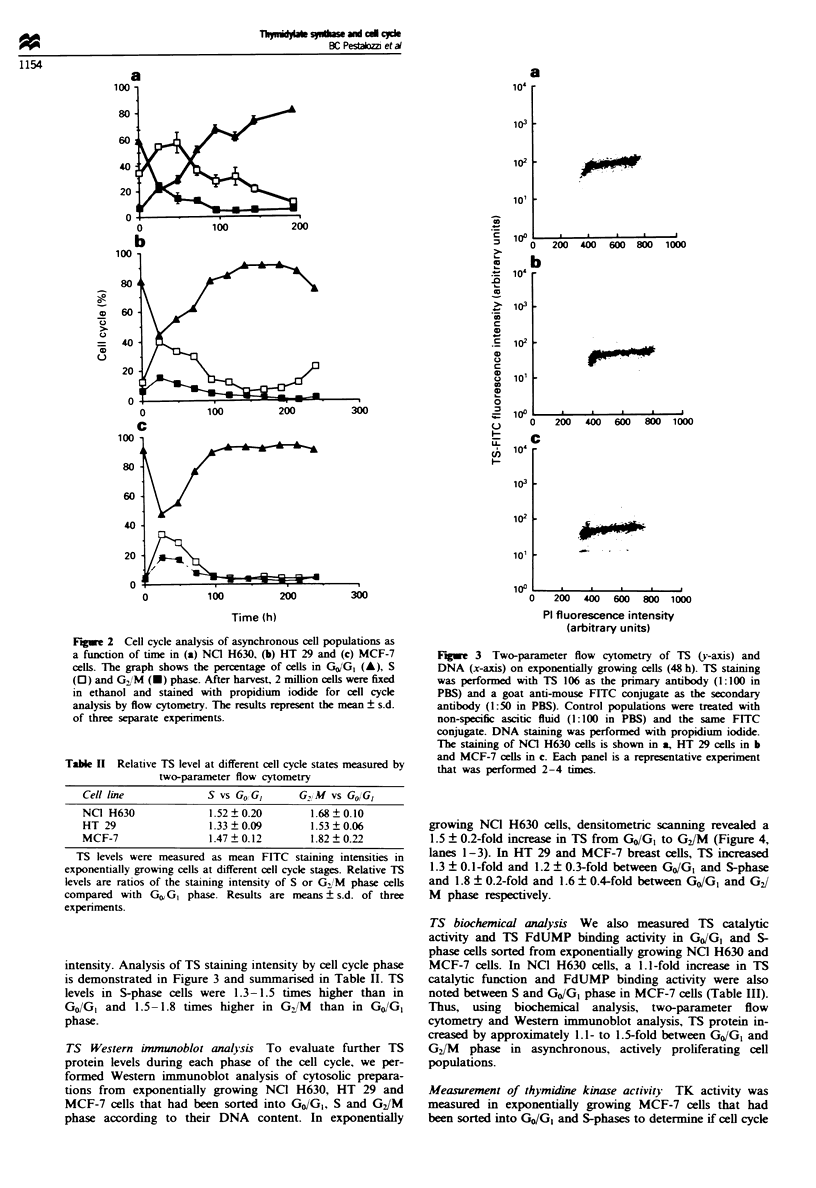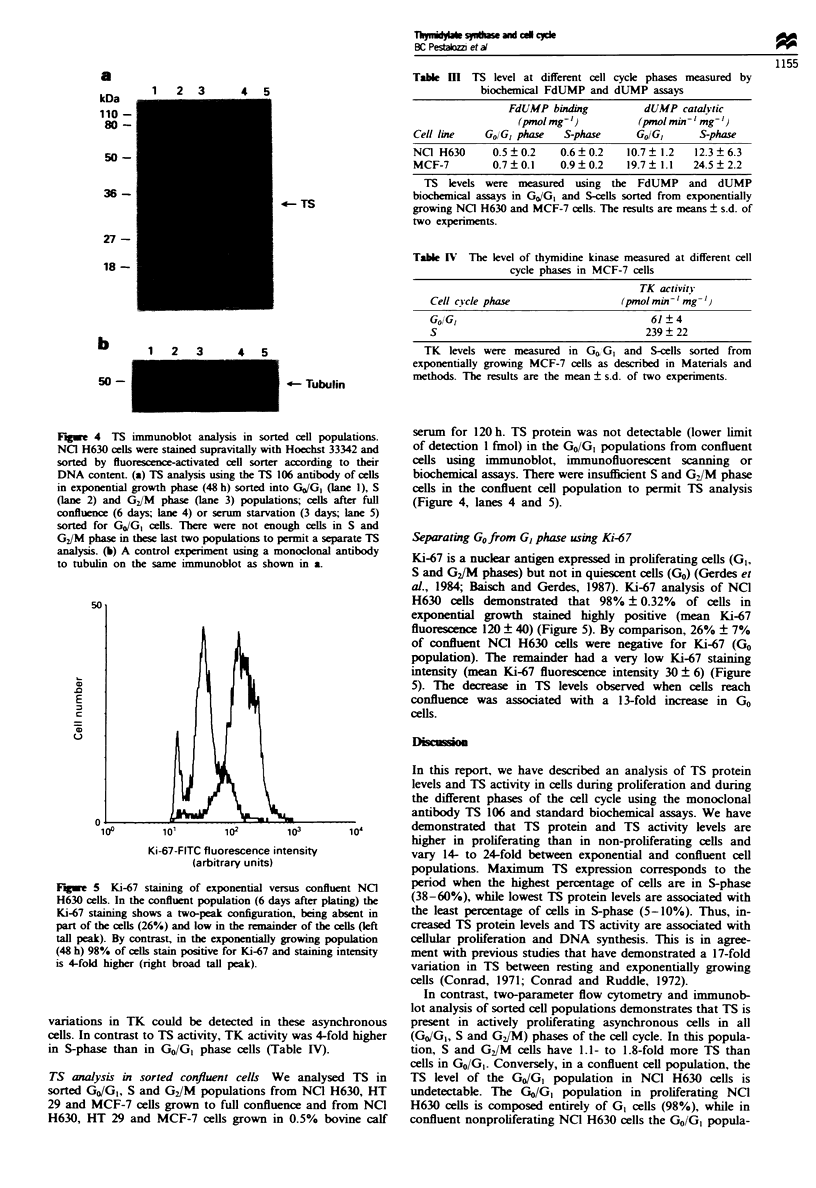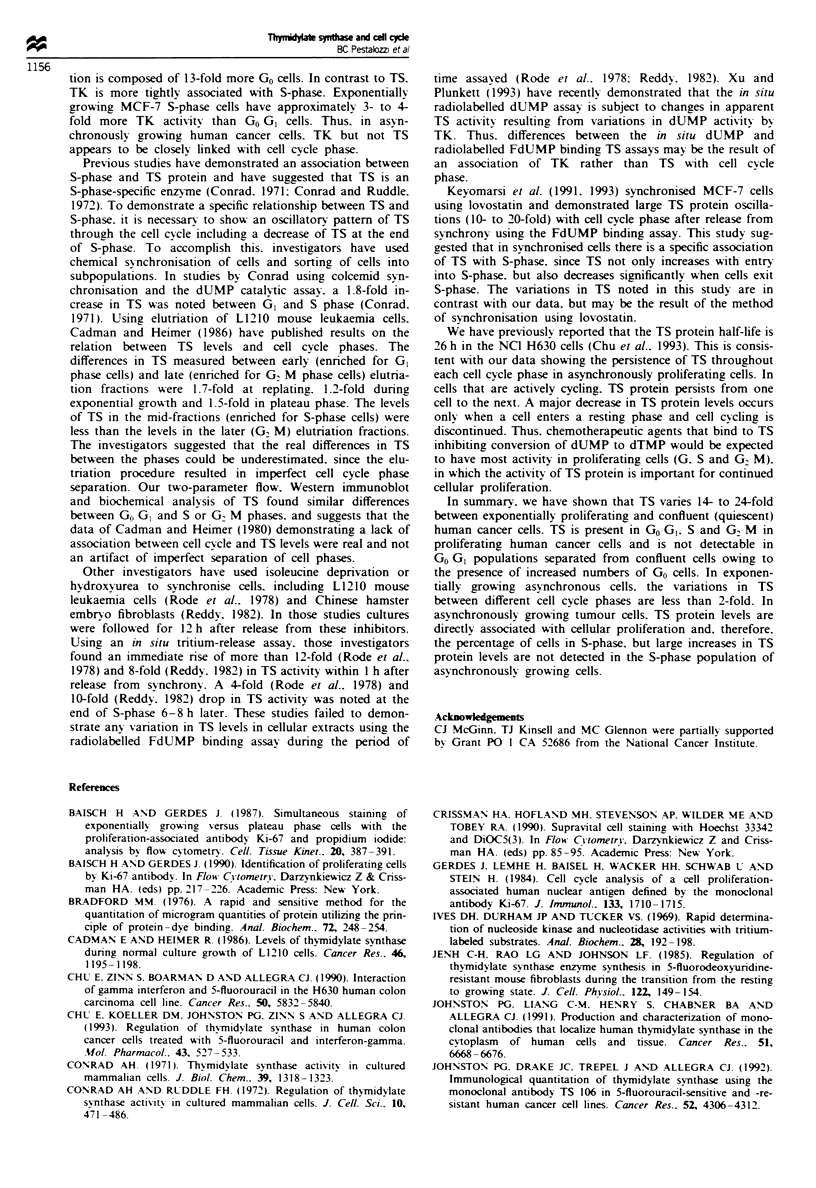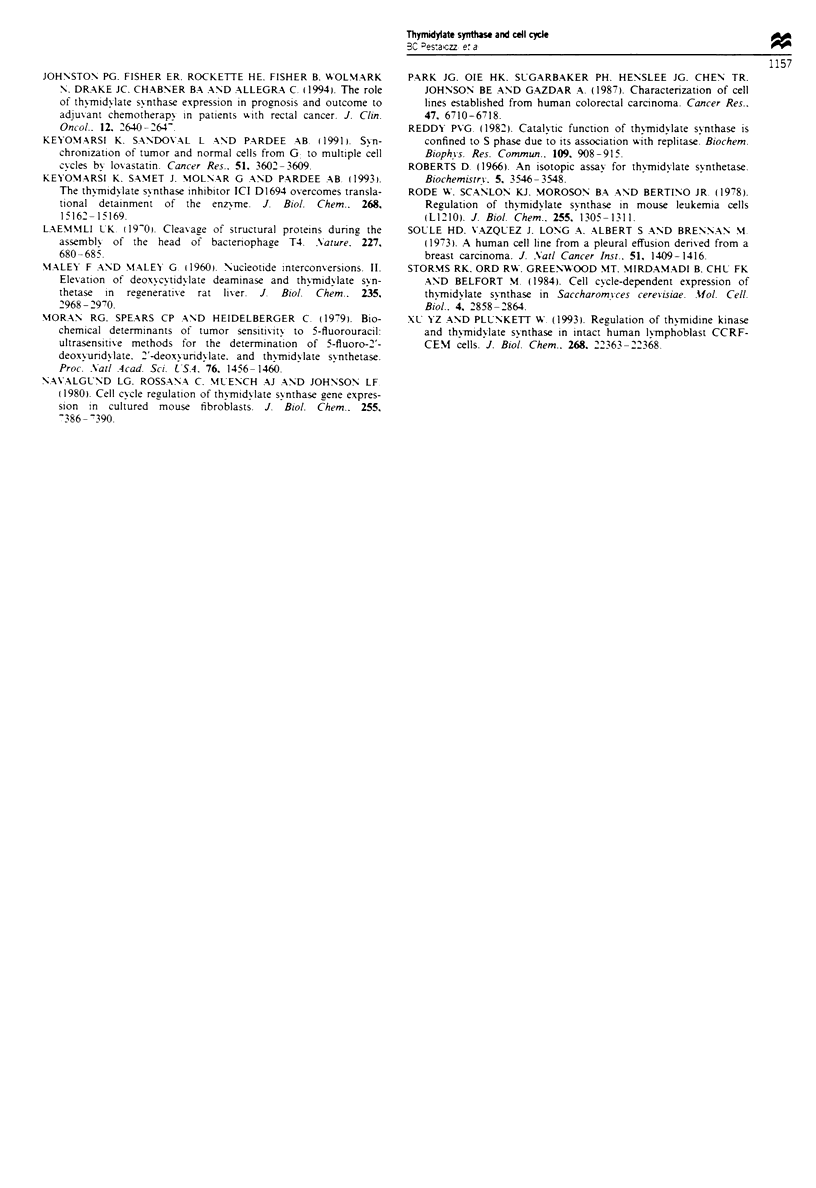# Increased thymidylate synthase protein levels are principally associated with proliferation but not cell cycle phase in asynchronous human cancer cells.

**DOI:** 10.1038/bjc.1995.225

**Published:** 1995-06

**Authors:** B. C. Pestalozzi, C. J. McGinn, T. J. Kinsella, J. C. Drake, M. C. Glennon, C. J. Allegra, P. G. Johnston

**Affiliations:** NCl-Navy Medical Oncology Branch, Bethesda, Maryland 20889-5105, USA.

## Abstract

**Images:**


British Journal of Cancer (1995) 71, 1151-1157

© 1995 Stockton Press All rights reserved 0007-0920/95 $12.00

### Increased thymidylate synthase protein levels are principally associated with proliferation but not cell cycle phase in asynchronous human cancer cells

BC Pestalozzi[1], CJ McGinn[2], TJ Kinsella[2], JC Drake[1], MC Glennon[2], CJ Allegra[1] and PG Johnston[1]

[1]*NCI-Navy Medical Oncology Branch, National Cancer Institute, Bethesda, Maryland 20889-5105 and* [2]*Department of Human Oncology, University of Wisconsin, Madison, Wisconsin 53792, USA.*

**Summary** We have analysed cell cycle variations in thymidylate synthase (TS) protein in asynchronously growing NCI H630 and HT 29 colon cancer and MCF-7 breast cancer cell lines. Western immunoblot analysis using the TS 106 monoclonal antibody revealed a 14- to 24-fold variation in TS levels between the peak exponential and confluent growth phase in the three cell lines. Similar variations in TS levels and TS activity were detected using the 5-fluorodeoxyuridine monophosphate and deoxyuridine monophosphate biochemical assays. The percentage of cells in S-phase, which paralleled changes in TS levels, reached a maximum of 38-60% in asynchronous exponentially growing cells compared with 5-10% in confluent cells. In asynchronous exponential cells, analysis of TS levels in each cell cycle phase using two-parameter flow cytometric analysis revealed that TS protein levels were 1.3- to 1.5-fold higher in S than in $G_0$ $G_1$ phase cells, and 1.5- to 1.8-fold higher in $G_2$ M than $G_0$ $G_1$ cells. Similar differences of 1.1- to 1.5-fold between $G_0$ $G_1$ and S-phase and 1.6- to 1.9-fold between $G_0$ $G_1$ and $G_2$ M-phase were detected by Western immunoblot and biochemical assays. TS protein was not detectable by Western blot analysis, flow cytometry or biochemical analysis in the $G_0$ $G_1$ population of confluent cells. Twenty-six per cent of cells in this population were $G_0$ cells compared with 2% in exponentially growing cells. In contrast to TS, a 4-fold difference in thymidine kinase (TK) was detected between $G_0$ $G_1$ and S-phase cells in exponentially growing MCF-7 cells. The level of TS enzyme is associated with cellular proliferation and the percentage of cells in S-phase; however, TS protein is not exclusively associated with S-phase in asynchronously growing cells. The variation in TS levels between exponentially growing and confluent cell population appears to be due to differences in TS levels between $G_0$ and $G_1$ cells.

**Keywords:** cell cycle; TS

Thymidylate synthase (TS; EC 2.1.1.45) catalyses the reductive methylation of deoxyuridine monophosphate (dUMP) to deoxythymidine monophosphate (dTMP). This reaction is an essential step in DNA biosynthesis, since it provides the only *de novo* source of thymidylate. TS is also a critical target for the fluoropyrimidine drugs that are widely used in the treatment of breast cancer, as well as tumours of the gastrointestinal and upper aerodigestive tracts. In tumour cells, 5-fluorouracil (5-FU) and fluorodeoxyuridine (FdUrd) are converted to 5-fluorodeoxyuridine monophosphate (FdUMP), which forms a covalent ternary complex with TS in the presence of the folate co-factor 5, 10-methylene tetrahydrofolate (5, 10-$CH_2H_4$PteGlu).

Previous studies have demonstrated in both mammalian and yeast systems that TS activity is higher during DNA replication and decreases when cells are non-dividing (Conrad, 1971; Maley and Maley, 1960; Navalgund *et al.*, 1980; Storms *et al.*, 1984). Other studies have shown that, while increased TS activity correlates with the DNA synthetic phase, this increase is not blocked by inhibitors of DNA synthesis (Jenh *et al.*, 1985). This suggests that, while TS activity may be associated with proliferation, its regulation may be independent of DNA synthesis and cell cycle phase. More recent studies have demonstrated that TS enzyme levels rise acutely when cells are exposed to cytotoxic agents such as 5-FU (Chu *et al.*, 1990). Thus, in addition to changes in TS related to the cell cycle, neoplastic cells may increase TS levels as a protective mechanism against cytotoxic stress. This acute induction of TS protein may represent an important mechanism in the development of tumour resistance.

Recently, we have developed several monoclonal antibodies to human TS that are highly specific and detect TS in the cytoplasm of tumour cells and tissue (Johnston *et al.*, 1991, 1992). These antibodies have facilitated the study of TS in cell lines and human tissues and have allowed TS to be measured within individual cells. We have also demonstrated that increased TS protein levels predict for poor clinical outcome in patients with rectal cancer (Johnston *et al.*, 1994). This may be the result of the association of TS protein levels with cellular proliferation.

The purpose of this study was to analyse cell cycle variations in TS levels during the various cell cycle phases and proliferation to determine its association with DNA synthesis and S-phase in asynchronously growing tumour cells.

### Materials and methods

### *Chemicals*

Dextran (clinical grade), 5-FU, acid-washed activated charcoal, fluorescein isothiocyanate (FITC)-labelled goat anti-mouse conjugate, propidium iodide, cycloheximide, thimerosal and non-specific murine ascitic fluid were all purchased from Sigma, (St Louis, MO, USA). [6-$^3$H]5-FdUMP (specific activity 23 Ci $mmol^{-1}$ and [5-$^3$H]dUMP (sp. act. 22 Ci $mmol^{-1}$) were obtained from Moravek Biochemicals (Brea, CA, USA). The ECL-enhanced chemiluminescence kit was obtained from Amersham (Buckinghamshire, UK). Nitrocellulose membranes were purchased from Schleicher & Schuell (Keene, NH, USA). The monoclonal antibody to α-tubulin was obtained from Oncogene Science (Uniondale, NY, USA). Ki-67 monoclonal antibody was obtained from Dako (Carpinteria, CA, USA). Goat anti-mouse horseradish

Correspondence: PG Johnston, NCI-Navy Medical Oncology Branch, National Cancer Institute, Naval Hospital Bethesda, 8901 Wisconsin Ave, Building 8, Room 5101, Bethesda MD 20889-5105, USA
Received 19 October 1994; revised 1 February 1995; accepted 2 February 1995

peroxidase conjugate, BioRad protein assay, Tween 20 and SDS were obtained from Bio-Rad Laboratories (Richmond, CA, USA). Tris and glycine were purchased from Schwarz/Mann Biotech, ICN Biomedicals (Cleveland, OH, USA). Phosphate-buffered saline (PBS) was obtained from Digene Diagnostics (Beltsville, MD, USA). Acrylamide was purchased from National Diagnostics (Atlanta, GA, USA). Ammonium persulphate was bought from BRL Life Technologicals (Gaithersburg, MD, USA). Carnation non-fat milk was obtained from Carnation (Los Angeles, CA, USA).

*Cell culture*

The characteristics of the human colon cancer cell lines NCI H630 and HT 29 and the human breast cancer cell line MCF-7 have been previously described (Park *et al.*, 1987; Soule *et al.*, 1973). All cells were maintained in RPMI-1640 (Gibco, Grand Island, NY) with 10% heat-inactivated fetal calf serum (FCS) plus 2 mM glutamine. For proliferation assays, cells were seeded on day 0 at a density of $2 \times 10^4$ cells $cm^{-2}$. Doubling times were calculated over the first 72 h of cell growth using linear regression analysis.

*Cell harvest*

Cells were trypsinised, resuspended in RPMI medium containing 10% FCS, and an aliquot was counted using a ZF Coulter Counter (Coulter Electronics, Hialeah, FL, USA). Cells were washed twice in ice-cold PBS and stored as pellets at −80°C. Before analysis, cells were lysed by sonication in 0.1 M potassium dihydrogen phosphate pH 7.4. Sonication of $1 \times 10^6$ cells $ml^{-1}$ was performed with four 2-s bursts from a Vibra cell sonifier from Sonics and Materials (Danbury, CT, USA). The cellular extracts were centrifuged at 5000 *g* for 15 min, and the supernatants collected. Protein concentrations were determined using the BioRad protein assay (Bradford, 1976).

*Western blot analysis*

Equivalent amounts of protein (400 μg) from each cellular lysate were resolved by polyacrylamide gel electrophoresis using 12.5% acrylamide, according to the method of Laemmli (1970). The gels were electroblotted onto a nitrocellulose membrane in transfer buffer (48 mM Tris, 39 mM glycine, 0.5 M EDTA in 20% methanol) for 2 h. The nitrocellulose blots were treated at room temperature with blocking solution (blotto: 5% Carnation non-fat milk, 10 mM Tris, 0.01% thimerosal) for 45 min. After washing with PSB–Tween (PBS with 0.1% Tween 20), primary antibody (TS 106, ascitic fluid, 1:100 in blotto) was added for 90 min. After two washes with PBS–Tween and three washes with blotto, secondary antibody (goat anti-mouse horseradish peroxidase, BioRad, 1:1000 in blotto) was used for 60 min. After another four washes with PBS–Tween, the chemiluminescent substrate (luminol, plus enhancer, according to the ECL method of Amersham) was applied for 1 min. Blots were then air dried, covered by a plastic foil and exposed to film (Kodak, X-OmatAR) for 5–60 s. Densitometry scanning of the film was performed using a Hewlett-Packard Scan Jet Plus and analysed using an image analysis software program (NIH IMAGE v.1.40; provided by Wayne Rasband, NIMH, NIH, Bethesda, MD, USA).

*FdUMP binding assay*

Equivalent volumes of cytosolic extracts (50 μl) were assayed in duplicate. The assay was performed in a total volume of 200 μl containing 75 μM 5, 10-$CH_2H_4$PteGlu, 3 pmol of [$^3$H]FdUMP, 100 mM 2-mercaptoethanol and 50 mM potassium dihydrogen phosphate pH 7.4 as has been previously described (Moran *et al.*, 1979; Johnston *et al.*, 1991).

*dUMP catalytic activity*

Equivalent volumes of cytosolic extracts (50 μl) were assayed in duplicate. The assay was performed in a final volume of 200 μl containing 100 pmol [5-$^3$H]dUMP, 100 mM 2-mercaptoethanol, 50 mM potassium dihydrogen phosphate pH 7.2 and 50 μl (or 5 μl) of cellular extracts as previously described (Roberts, 1966).

*Thymidine kinase assay*

Thymidine kinase was assayed as previously described by Ives *et al.* (1969). The reaction mixture consisted of 10 mM ATP, 10 mM magnesium chloride 50 mM Tris-HCl pH 7.5, 15 mM sodium fluoride 0.1 μCi [$^3$H]thymidine (Moravek 20 Ci $mmol^{-1}$), 5 μM unlabelled thymidine, 1–20 μl of cell lysate in a total volume of 50 μl. The reaction was allowed to proceed for 30 min at room temperature and stopped by boiling for 60 s. The reaction as measured was linear with time, and the rate was proportional to the lysate volume used. The assay mixture was spotted onto a 2.5 cm DE 81 ion-exchange disc (Whatman). After 10 min the disc was washed with three changes of distilled water, 30 ml per disc, and then placed in scintillation vials containing 1 ml of 0.1 M hydrochloric acid–0.2 M potassium chloride, and the vials were gently shaken for 20 min. Scintillation fluid was added and samples counted in a scintillation counter. The values were expressed as pmol $min^{-1}$ $mg^{-1}$ cytosol protein.

*Cell cycle distribution*

One to two million cells were resuspended in 0.7 ml of ice-cold PBS and fixed by adding 1.3 ml of 95% ethanol with 0.5% Tween 20 drop wise to the cell suspension with gentle vortex mixing. The cell suspension was kept at 4°C overnight. Cells were washed once in PBS and resuspended in 0.5 ml of PBS-TB [PBS with 0.1% (w/v) bovine serum albumin and 0.5% Tween 20] containing 10 μg $ml^{-1}$ RNAse and incubated at 37°C for 20 min. Cells were then pelleted and resuspended in 0.5 ml of PBS–TB containing 50 μg $ml^{-1}$ propidium iodide (PI). Cell cycle data were acquired using a Becton-Dickinson FACScan with 15 mW excitation at 488 nM. The PI signal was assessed through a 650 long-pass filter on FL3. The software used for acquisition and analysis was the CellFIT cell cycle analysis program version 2.01.2 from Becton-Dickinson Immunocytometry Systems (San Jose, CA, USA).

*Two-parameter flow cytometry with TS and PI*

Aliquots of two million cells were harvested by centrifugation at 500 *g*, washed and fixed as above. Cells were placed in blocking buffer BSA-T (3% BSA, 0.2% Tween 20 in PBS) at 4°C for 30 min. After pelleting, cells were incubated with 200 μl of the primary antibody (TS 106 monoclonal or non-specific ascitic fluid as a control at 1:100 dilution in PBS) for at least 1 hr at 4°C. After one wash, cells were incubated with 200 μl of the secondary antibody: goat anti-mouse immunoglobulin–fluorescein isothiocyanate (FITC) conjugate diluted 1:50 in PBS-TG [PBS with 0.1% (v/v) goat serum and 0.5% Tween 20]. After two washes, samples were resuspended in 0.5 ml of PBS-TB containing 50 μg $ml^{-1}$ of PI. The PI signal was assessed as described above. The FITC signal was collected through a 530/30 bandpass filter on FL1. Analysis was performed with the LYSYS II software version 1.1 from Becton-Dickinson. The analysis was restricted to singlets using pulse area *vs* pulse width gating of the PI signal. A DNA histogram was obtained from each sample and boundaries were established based on the mean channel of the $G_1$ and $G_2$/M peaks to define $G_0$/$G_1$, S-phase and $G_2$/M regions. In both the control population (ascitic fluid diluted 1:100 in PBS) and the population incubated with TS 106, the mean fluorescent intensity (MFI) of the FITC signal was determined from each phase of the cell cycle. Ratios of relative TS

staining intensities between different cell cycle phases were then calculated, after correcting for non-specific staining.

*Cell cycle sorting using Hoechst 33342*

Unfixed cells were stained with the supervital stain Hoechst 33342 according to the method of Crissman *et al.* (1990). Cells in $G_0/G_1$, S and $G_2/M$ were then sorted according to their DNA content, using a Becton-Dickinson FACStar Plus flow cytometer with 100 mW excitation at 351–465 nm. The Hoechst 33342 signal was assessed through a 400 long-pass filter into three distinct populations: $G_0/G_1$ phase, S-phase and $G_2/M$-phase cells. Pellets were stored at −80°C until assayed for TS protein levels and TS biochemical activity. Sorted populations were confirmed to contain only $G_0/G_1$, S or $G_2/M$ cells by subsequent cell cycle analysis with PI.

**Figure 1** TS levels in asynchronous populations of three human cancer cell lines. TS levels were measured by three different methods as a function of time: immunoblot analysis (■), FdUMP binding assay (O) and dUMP catalytic activity (▲). The three graphs display results for NCI H630 (**a**), HT 29 (**b**) and MCF-7 (**c**) cells. Cells were plated at $2 \times 10^4$ cells $cm^{-2}$ and grown for up to 288 h. After harvest, crude cellular extracts were made by sonication and aliquots were analysed in parallel by immunoblot, FdUMP binding and dUMP catalytic assays respectively. The results represent the mean ± s.d. of three separate experiments.

*Two-parameter flow cytometry with Ki-67 and PI*

This assay was based on a previously published method (Baisch and Gerdes, 1990). After harvest, one million cells were resuspended in 2 ml of 0.15 M sodium chloride at 4°C. The suspension was added dropwise to 8 ml of ice-cold pure acetone while gently shaking. Cells were stored for at least 24 h at −20°C. After centrifugation and decanting, 0.1% RNAse in PBS was added and the sample incubated for 20 min at 37°C. After pelleting, 100 μl of Ki-67 antibody (1:10 dilution in PBS containing 1% BSA) was added. Incubation lasted 30 min at room temperature with gentle shaking. Subsequently, 100 μl of FITC-conjugated goat anti-mouse antibody (1:40 in PBS–BSA) was added and incubated for 30 min at room temperature. After washing in PBS, 0.5 ml of PI (2 μg $ml^{-1}$ in PBS) was added for 20 min in the dark. Data were acquired and analysed as described above. The control populations in these experiments were incubated with non-specific mouse ascitic fluid rather than the Ki-67 antibody. The baseline fluorescence of the log-amplified Ki-67 FITC signal was set using the appropriate negative control for each sample. Peak fluorescence on Fl1 of this population was used to determine the upper boundary for cells considered to be Ki-67 negative.

### Results

*TS and cell cycle analysis during asynchronous growth*

NCI H630, HT 29 and MCF-7 cells were seeded at an equivalent density ($10^4$ cells $cm^{-2}$). The doubling time for NCI H630 cells was 25 h, for HT 29 cells 22 h and for MCF-7 cells 19 h. TS levels were measured at various time points during asynchronous growth (hours 0, 24, 48, 72, 96, 120, 144, 168, 192, 240 and 288). Immunoblot analysis revealed a 14- to 24-fold variation in TS protein levels from peak exponential growth phase to confluent growth phase in the three cell lines. TS levels were maximal after 48 h of growth and reached the lowest level after 120 h (Figure 1). The drop in TS from the maximal to the lowest basal level occurred over a brief period of time (48 h) between hours 72 and 120. Similar variations in TS protein levels and TS activity over time were also detected using the FdUMP binding and the dUMP catalytic assays respectively (Figure 1). Comparing all three methods, the differences between the maximum and minimum TS level were 14- to 17-fold in the H630 colon cancer cell line, 15- to 23-fold in the HT 29 colon cancer cell line and 19- to 24-fold in the MCF-7 breast cancer cell line (Table 1). The change in TS levels was paralleled by similar variations in the distribution of cells through the cell cycle (Figure 2). The peak percentage of S-phase cells was reached after 24–48 h of growth and decreased 6- to 10-fold after 120 h. Thus, increased TS levels were associated with increased DNA synthesis.

*TS analysis by cell cycle phase*

*Two-parameter flow cytometry and cell sorting* Exponentially growing asynchronous NCI H630, HT 29 and MCF-7 cells were analysed by two-parameter flow cytometry for TS protein and DNA content. TS was measured as FITC fluorescence intensity and DNA content as PI fluorescence

**Table 1** TS levels in confluent *vs* exponentially growing cells

| Cell line | *Immunoblot relative density* *Variation*[a] | *FdUMP Assay (pmol mg⁻¹)* *Maximum*[b] | *Variation*[a] | *dUMP assay (pmol min⁻¹ mg⁻¹)* *Maximum*[b] | *Variation*[a] |
|---|---|---|---|---|---|
| H630 | 14-fold | 0.675 ± 0.04 | 17-fold | 6.89 ± 0.2 | 15-fold |
| HT 29 | 23-fold | 0.269 ± 0.01 | 15-fold | 4.84 ± 0.3 | 19-fold |
| MCF-7 | 22-fold | 0.809 ± 0.02 | 24-fold | 13.7 ± 0.5 | 19-fold |

[a]Variation between maximum level of mean TS (exponential growth phase) and mean basal level of TS (plateau phase, hours 120–288). [b]Maximum level of mean TS measured during exponential growth phase.

**Figure 2** Cell cycle analysis of asynchronous cell populations as a function of time in (**a**) NCI H630, (**b**) HT 29 and (**c**) MCF-7 cells. The graph shows the percentage of cells in $G_0/G_1$ (▲), S (□) and $G_2/M$ (■) phase. After harvest, 2 million cells were fixed in ethanol and stained with propidium iodide for cell cycle analysis by flow cytometry. The results represent the mean ± s.d. of three separate experiments.

**Figure 3** Two-parameter flow cytometry of TS (*y*-axis) and DNA (*x*-axis) on exponentially growing cells (48 h). TS staining was performed with TS 106 as the primary antibody (1:100 in PBS) and a goat anti-mouse FITC conjugate as the secondary antibody (1:50 in PBS). Control populations were treated with non-specific ascitic fluid (1:100 in PBS) and the same FITC conjugate. DNA staining was performed with propidium iodide. The staining of NCI H630 cells is shown in **a**, HT 29 cells in **b** and MCF-7 cells in **c**. Each panel is a representative experiment that was performed 2–4 times.

**Table II** Relative TS level at different cell cycle states measured by two-parameter flow cytometry

| *Cell line* | *S* vs $G_0/G_1$ | $G_2/M$ vs $G_0/G_1$ |
|---|---|---|
| NCI H630 | 1.52 ± 0.20 | 1.68 ± 0.10 |
| HT 29 | 1.33 ± 0.09 | 1.53 ± 0.06 |
| MCF-7 | 1.47 ± 0.12 | 1.82 ± 0.22 |

TS levels were measured as mean FITC staining intensities in exponentially growing cells at different cell cycle stages. Relative TS levels are ratios of the staining intensity of S or $G_2/M$ phase cells compared with $G_0/G_1$ phase. Results are means ± s.d. of three experiments.

intensity. Analysis of TS staining intensity by cell cycle phase is demonstrated in Figure 3 and summarised in Table II. TS levels in S-phase cells were 1.3–1.5 times higher than in $G_0/G_1$ and 1.5–1.8 times higher in $G_2/M$ than in $G_0/G_1$ phase.

*TS Western immunoblot analysis* To evaluate further TS protein levels during each phase of the cell cycle, we performed Western immunoblot analysis of cytosolic preparations from exponentially growing NCI H630, HT 29 and MCF-7 cells that had been sorted into $G_0/G_1$, S and $G_2/M$ phase according to their DNA content. In exponentially growing NCI H630 cells, densitometric scanning revealed a 1.5 ± 0.2-fold increase in TS from $G_0/G_1$ to $G_2/M$ (Figure 4, lanes 1–3). In HT 29 and MCF-7 breast cells, TS increased 1.3 ± 0.1-fold and 1.2 ± 0.3-fold between $G_0/G_1$ and S-phase and 1.8 ± 0.2-fold and 1.6 ± 0.4-fold between $G_0/G_1$ and $G_2/M$ phase respectively.

*TS biochemical analysis* We also measured TS catalytic activity and TS FdUMP binding activity in $G_0/G_1$ and S-phase cells sorted from exponentially growing NCI H630 and MCF-7 cells. In NCI H630 cells, a 1.1-fold increase in TS catalytic function and FdUMP binding activity were also noted between S and $G_0/G_1$ phase in MCF-7 cells (Table III). Thus, using biochemical analysis, two-parameter flow cytometry and Western immunoblot analysis, TS protein increased by approximately 1.1- to 1.5-fold between $G_0/G_1$ and $G_2/M$ phase in asynchronous, actively proliferating cell populations.

*Measurement of thymidine kinase activity* TK activity was measured in exponentially growing MCF-7 cells that had been sorted into $G_0/G_1$ and S-phases to determine if cell cycle

**Figure 4** TS immunoblot analysis in sorted cell populations. NCl H630 cells were stained supravitally with Hoechst 33342 and sorted by fluorescence-activated cell sorter according to their DNA content. (**a**) TS analysis using the TS 106 antibody of cells in exponential growth phase (48 h) sorted into $G_0/G_1$ (lane 1), S (lane 2) and $G_2/M$ phase (lane 3) populations; cells after full confluence (6 days; lane 4) or serum starvation (3 days; lane 5) sorted for $G_0/G_1$ cells. There were not enough cells in S and $G_2/M$ phase in these last two populations to permit a separate TS analysis. (**b**) A control experiment using a monoclonal antibody to tubulin on the same immunoblot as shown in **a**.

**Figure 5** Ki-67 staining of exponential versus confluent NCl H630 cells. In the confluent population (6 days after plating) the Ki-67 staining shows a two-peak configuration, being absent in part of the cells (26%) and low in the remainder of the cells (left tall peak). By contrast, in the exponentially growing population (48 h) 98% of cells stain positive for Ki-67 and staining intensity is 4-fold higher (right broad tall peak).

variations in TK could be detected in these asynchronous cells. In contrast to TS activity, TK activity was 4-fold higher in S-phase than in $G_0/G_1$ phase cells (Table IV).

*TS analysis in sorted confluent cells* We analysed TS in sorted $G_0/G_1$, S and $G_2/M$ populations from NCl H630, HT 29 and MCF-7 cells grown to full confluence and from NCl H630, HT 29 and MCF-7 cells grown in 0.5% bovine calf serum for 120 h. TS protein was not detectable (lower limit of detection 1 fmol) in the $G_0/G_1$ populations from confluent cells using immunoblot, immunofluorescent scanning or biochemical assays. There were insufficient S and $G_2/M$ phase cells in the confluent cell population to permit TS analysis (Figure 4, lanes 4 and 5).

**Table III** TS level at different cell cycle phases measured by biochemical FdUMP and dUMP assays

| | *FdUMP binding (pmol mg$^{-1}$)* | | *dUMP catalytic (pmol min$^{-1}$ mg$^{-1}$)* | |
|---|---|---|---|---|
| *Cell line* | *$G_0/G_1$ phase* | *S-phase* | *$G_0/G_1$* | *S-phase* |
| NCl H630 | 0.5 ± 0.2 | 0.6 ± 0.2 | 10.7 ± 1.2 | 12.3 ± 6.3 |
| MCF-7 | 0.7 ± 0.1 | 0.9 ± 0.2 | 19.7 ± 1.1 | 24.5 ± 2.2 |

TS levels were measured using the FdUMP and dUMP biochemical assays in $G_0/G_1$ and S-cells sorted from exponentially growing NCl H630 and MCF-7 cells. The results are means ± s.d. of two experiments.

**Table IV** The level of thymidine kinase measured at different cell cycle phases in MCF-7 cells

| *Cell cycle phase* | *TK activity (pmol min$^{-1}$ mg$^{-1}$)* |
|---|---|
| *$G_0/G_1$* | *61 ± 4* |
| *S* | 239 ± 22 |

TK levels were measured in $G_0/G_1$ and S-cells sorted from exponentially growing MCF-7 cells as described in Materials and methods. The results are the mean ± s.d. of two experiments.

### *Separating $G_0$ from $G_1$ phase using Ki-67*

Ki-67 is a nuclear antigen expressed in proliferating cells ($G_1$, S and $G_2/M$ phases) but not in quiescent cells ($G_0$) (Gerdes *et al.*, 1984; Baisch and Gerdes, 1987). Ki-67 analysis of NCl H630 cells demonstrated that 98% ± 0.32% of cells in exponential growth stained highly positive (mean Ki-67 fluorescence 120 ± 40) (Figure 5). By comparison, 26% ± 7% of confluent NCl H630 cells were negative for Ki-67 ($G_0$ population). The remainder had a very low Ki-67 staining intensity (mean Ki-67 fluorescence intensity 30 ± 6) (Figure 5). The decrease in TS levels observed when cells reach confluence was associated with a 13-fold increase in $G_0$ cells.

### Discussion

In this report, we have described an analysis of TS protein levels and TS activity in cells during proliferation and during the different phases of the cell cycle using the monoclonal antibody TS 106 and standard biochemical assays. We have demonstrated that TS protein and TS activity levels are higher in proliferating than in non-proliferating cells and vary 14- to 24-fold between exponential and confluent cell populations. Maximum TS expression corresponds to the period when the highest percentage of cells are in S-phase (38–60%), while lowest TS protein levels are associated with the least percentage of cells in S-phase (5–10%). Thus, increased TS protein levels and TS activity are associated with cellular proliferation and DNA synthesis. This is in agreement with previous studies that have demonstrated a 17-fold variation in TS between resting and exponentially growing cells (Conrad, 1971; Conrad and Ruddle, 1972).

In contrast, two-parameter flow cytometry and immunoblot analysis of sorted cell populations demonstrates that TS is present in actively proliferating asynchronous cells in all ($G_0/G_1$, S and $G_2/M$) phases of the cell cycle. In this population, S and $G_2/M$ cells have 1.1- to 1.8-fold more TS than cells in $G_0/G_1$. Conversely, in a confluent cell population, the TS level of the $G_0/G_1$ population in NCl H630 cells is undetectable. The $G_0/G_1$ population in proliferating NCl H630 cells is composed entirely of $G_1$ cells (98%), while in confluent nonproliferating NCl H630 cells the $G_0/G_1$ population is composed of 13-fold more $G_0$ cells. In contrast to TS, TK is more tightly associated with S-phase. Exponentially growing MCF-7 S-phase cells have approximately 3- to 4-fold more TK activity than $G_0G_1$ cells. Thus, in asynchronously growing human cancer cells, TK but not TS appears to be closely linked with cell cycle phase.

Previous studies have demonstrated an association between S-phase and TS protein and have suggested that TS is an S-phase-specific enzyme (Conrad, 1971; Conrad and Ruddle, 1972). To demonstrate a specific relationship between TS and S-phase, it is necessary to show an oscillatory pattern of TS through the cell cycle including a decrease of TS at the end of S-phase. To accomplish this, investigators have used chemical synchronisation of cells and sorting of cells into subpopulations. In studies by Conrad using colcemid synchronisation and the dUMP catalytic assay, a 1.8-fold increase in TS was noted between $G_1$ and S phase (Conrad, 1971). Using elutriation of L1210 mouse leukaemia cells, Cadman and Heimer (1986) have published results on the relation between TS levels and cell cycle phases. The differences in TS measured between early (enriched for $G_1$ phase cells) and late (enriched for $G_2M$ phase cells) elutriation fractions were 1.7-fold at replating, 1.2-fold during exponential growth and 1.5-fold in plateau phase. The levels of TS in the mid-fractions (enriched for S-phase cells) were less than the levels in the later ($G_2M$) elutriation fractions. The investigators suggested that the real differences in TS between the phases could be underestimated, since the elutriation procedure resulted in imperfect cell cycle phase separation. Our two-parameter flow, Western immunoblot and biochemical analysis of TS found similar differences between $G_0G_1$ and S or $G_2M$ phases, and suggests that the data of Cadman and Heimer (1980) demonstrating a lack of association between cell cycle and TS levels were real and not an artifact of imperfect separation of cell phases.

Other investigators have used isoleucine deprivation or hydroxyurea to synchronise cells, including L1210 mouse leukaemia cells (Rode *et al.*, 1978) and Chinese hamster embryo fibroblasts (Reddy, 1982). In those studies cultures were followed for 12 h after release from these inhibitors. Using an *in situ* tritium-release assay, those investigators found an immediate rise of more than 12-fold (Rode *et al.*, 1978) and 8-fold (Reddy, 1982) in TS activity within 1 h after release from synchrony. A 4-fold (Rode *et al.*, 1978) and 10-fold (Reddy, 1982) drop in TS activity was noted at the end of S-phase 6–8 h later. These studies failed to demonstrate any variation in TS levels in cellular extracts using the radiolabelled FdUMP binding assay during the period of time assayed (Rode *et al.*, 1978; Reddy, 1982). Xu and Plunkett (1993) have recently demonstrated that the *in situ* radiolabelled dUMP assay is subject to changes in apparent TS activity resulting from variations in dUMP activity by TK. Thus, differences between the *in situ* dUMP and radiolabelled FdUMP binding TS assays may be the result of an association of TK rather than TS with cell cycle phase.

Keyomarsi *et al.* (1991, 1993) synchronised MCF-7 cells using lovostatin and demonstrated large TS protein oscillations (10- to 20-fold) with cell cycle phase after release from synchrony using the FdUMP binding assay. This study suggested that in synchronised cells there is a specific association of TS with S-phase, since TS not only increases with entry into S-phase, but also decreases significantly when cells exit S-phase. The variations in TS noted in this study are in contrast with our data, but may be the result of the method of synchronisation using lovostatin.

We have previously reported that the TS protein half-life is 26 h in the NCl H630 cells (Chu *et al.*, 1993). This is consistent with our data showing the persistence of TS throughout each cell cycle phase in asynchronously proliferating cells. In cells that are actively cycling, TS protein persists from one cell to the next. A major decrease in TS protein levels occurs only when a cell enters a resting phase and cell cycling is discontinued. Thus, chemotherapeutic agents that bind to TS inhibiting conversion of dUMP to dTMP would be expected to have most activity in proliferating cells (G, S and $G_2M$), in which the activity of TS protein is important for continued cellular proliferation.

In summary, we have shown that TS varies 14- to 24-fold between exponentially proliferating and confluent (quiescent) human cancer cells. TS is present in $G_0G_1$, S and $G_2M$ in proliferating human cancer cells and is not detectable in $G_0G_1$ populations separated from confluent cells owing to the presence of increased numbers of $G_0$ cells. In exponentially growing asynchronous cells, the variations in TS between different cell cycle phases are less than 2-fold. In asynchronously growing tumour cells, TS protein levels are directly associated with cellular proliferation and, therefore, the percentage of cells in S-phase, but large increases in TS protein levels are not detected in the S-phase population of asynchronously growing cells.

**Acknowledgements**

CJ McGinn, TJ Kinsell and MC Glennon were partially supported by Grant PO 1 CA 52686 from the National Cancer Institute.

**References**

BAISCH H AND GERDES J. (1987). Simultaneous staining of exponentially growing versus plateau phase cells with the proliferation-associated antibody Ki-67 and propidium iodide: analysis by flow cytometry. *Cell. Tissue Kinet.*, **20**, 387–391.

BAISCH H AND GERDES J. (1990). Identification of proliferating cells by Ki-67 antibody. In *Flow Cytometry*, Darzynkiewicz Z & Crissman HA. (eds) pp. 217–226. Academic Press: New York.

BRADFORD MM. (1976). A rapid and sensitive method for the quantitation of microgram quantities of protein utilizing the principle of protein-dye binding. *Anal. Biochem.*, **72**, 248–254.

CADMAN E AND HEIMER R. (1986). Levels of thymidylate synthase during normal culture growth of L1210 cells. *Cancer Res.*, **46**, 1195–1198.

CHU E, ZINN S, BOARMAN D AND ALLEGRA CJ. (1990). Interaction of gamma interferon and 5-fluorouracil in the H630 human colon carcinoma cell line. *Cancer Res.*, **50**, 5832–5840.

CHU E, KOELLER DM, JOHNSTON PG, ZINN S AND ALLEGRA CJ. (1993). Regulation of thymidylate synthase in human colon cancer cells treated with 5-fluorouracil and interferon-gamma. *Mol. Pharmacol.*, **43**, 527–533.

CONRAD AH. (1971). Thymidylate synthase activity in cultured mammalian cells. *J. Biol. Chem.*, **39**, 1318–1323.

CONRAD AH AND RUDDLE FH. (1972). Regulation of thymidylate synthase activity in cultured mammalian cells. *J. Cell. Sci.*, **10**, 471–486.

CRISSMAN HA, HOFLAND MH, STEVENSON AP, WILDER ME AND TOBEY RA. (1990). Supravital cell staining with Hoechst 33342 and DiOC5(3). In *Flow Cytometry*, Darzynkiewicz Z and Crissman HA. (eds) pp. 85–95. Academic Press: New York.

GERDES J, LEMHE H, BAISEL H, WACKER HH, SCHWAB U AND STEIN H. (1984). Cell cycle analysis of a cell proliferation-associated human nuclear antigen defined by the monoclonal antibody Ki-67. *J. Immunol.*, **133**, 1710–1715.

IVES DH, DURHAM JP AND TUCKER VS. (1969). Rapid determination of nucleoside kinase and nucleotidase activities with tritium-labeled substrates. *Anal. Biochem.*, **28**, 192–198.

JENH C-H, RAO LG AND JOHNSON LF. (1985). Regulation of thymidylate synthase enzyme synthesis in 5-fluorodeoxyuridine-resistant mouse fibroblasts during the transition from the resting to growing state. *J. Cell. Physiol.*, **122**, 149–154.

JOHNSTON PG, LIANG C-M, HENRY S, CHABNER BA AND ALLEGRA CJ. (1991). Production and characterization of monoclonal antibodies that localize human thymidylate synthase in the cytoplasm of human cells and tissue. *Cancer Res.*, **51**, 6668–6676.

JOHNSTON PG, DRAKE JC, TREPEL J AND ALLEGRA CJ. (1992). Immunological quantitation of thymidylate synthase using the monoclonal antibody TS 106 in 5-fluorouracil-sensitive and -resistant human cancer cell lines. *Cancer Res.*, **52**, 4306–4312.

JOHNSTON PG. FISHER ER. ROCKETTE HE. FISHER B. WOLMARK N. DRAKE JC. CHABNER BA AND ALLEGRA C. (1994). The role of thymidylate synthase expression in prognosis and outcome to adjuvant chemotherapy in patients with rectal cancer. *J. Clin. Oncol.*, **12**, 2640-2647.

KEYOMARSI K. SANDOVAL L AND PARDEE AB. (1991). Synchronization of tumor and normal cells from $G_1$ to multiple cell cycles by lovastatin. *Cancer Res.*, **51**, 3602-3609.

KEYOMARSI K. SAMET J. MOLNAR G AND PARDEE AB. (1993). The thymidylate synthase inhibitor ICI D1694 overcomes translational detainment of the enzyme. *J. Biol. Chem.*, **268**, 15162-15169.

LAEMMLI UK. (1970). Cleavage of structural proteins during the assembly of the head of bacteriophage T4. *Nature*, **227**, 680-685.

MALEY F AND MALEY G. (1960). Nucleotide interconversions. II. Elevation of deoxycytidylate deaminase and thymidylate synthetase in regenerative rat liver. *J. Biol. Chem.*, **235**, 2968-2970.

MORAN RG. SPEARS CP AND HEIDELBERGER C. (1979). Biochemical determinants of tumor sensitivity to 5-fluorouracil: ultrasensitive methods for the determination of 5-fluoro-2'-deoxyuridylate, 2'-deoxyuridylate, and thymidylate synthetase. *Proc. Natl Acad. Sci. USA*, **76**, 1456-1460.

NAVALGUND LG. ROSSANA C. MUENCH AJ AND JOHNSON LF. (1980). Cell cycle regulation of thymidylate synthase gene expression in cultured mouse fibroblasts. *J. Biol. Chem.*, **255**, 7386-7390.

PARK JG. OIE HK. SUGARBAKER PH. HENSLEE JG. CHEN TR. JOHNSON BE AND GAZDAR A. (1987). Characterization of cell lines established from human colorectal carcinoma. *Cancer Res.*, **47**, 6710-6718.

REDDY PVG. (1982). Catalytic function of thymidylate synthase is confined to S phase due to its association with replitase. *Biochem. Biophys. Res. Commun.*, **109**, 908-915.

ROBERTS D. (1966). An isotopic assay for thymidylate synthetase. *Biochemistry*, **5**, 3546-3548.

RODE W. SCANLON KJ. MOROSON BA AND BERTINO JR. (1978). Regulation of thymidylate synthase in mouse leukemia cells (L1210). *J. Biol. Chem.*, **255**, 1305-1311.

SOULE HD. VAZQUEZ J. LONG A. ALBERT S AND BRENNAN M. (1973). A human cell line from a pleural effusion derived from a breast carcinoma. *J. Natl Cancer Inst.*, **51**, 1409-1416.

STORMS RK. ORD RW. GREENWOOD MT. MIRDAMADI B. CHU FK AND BELFORT M. (1984). Cell cycle-dependent expression of thymidylate synthase in *Saccharomyces cerevisiae*. *Mol. Cell. Biol.*, **4**, 2858-2864.

XU YZ AND PLUNKETT W. (1993). Regulation of thymidine kinase and thymidylate synthase in intact human lymphoblast CCRF-CEM cells. *J. Biol. Chem.*, **268**, 22363-22368.